# Fire Blight Susceptibility in *Lilium* spp. Correlates to Sensitivity to *Botrytis elliptica* Secreted Cell Death Inducing Compounds

**DOI:** 10.3389/fpls.2021.660337

**Published:** 2021-06-28

**Authors:** Michele C. Malvestiti, Richard G. H. Immink, Paul Arens, Thomas Quiroz Monnens, Jan A. L. van Kan

**Affiliations:** ^1^Laboratory of Phytopathology, Wageningen University & Research, Wageningen, Netherlands; ^2^Department of Bioscience, Wageningen University & Research, Wageningen, Netherlands; ^3^Laboratory of Molecular Biology, Wageningen University & Research, Wageningen, Netherlands; ^4^Department of Plant Breeding, Wageningen University & Research, Wageningen, Netherlands

**Keywords:** *Botrytis* fire blight, *Lilium*, effector proteins, necrotroph, programmed cell death

## Abstract

Fire blight represents a widespread disease in *Lilium* spp. and is caused by the necrotrophic Ascomycete *Botrytis elliptica*. There are >100 *Lilium* species that fall into distinct phylogenetic groups and these have been used to generate the contemporary commercial genotypes. It is known among lily breeders and growers that different groups of lilies differ in susceptibility to fire blight, but the genetic basis and mechanisms of susceptibility to fire blight are unresolved. The aim of this study was to quantify differences in fire blight susceptibility between plant genotypes and differences in virulence between fungal isolates. To this end we inoculated, in four biological replicates over 2 years, a set of 12 *B. elliptica* isolates on a panel of 18 lily genotypes representing seven *Lilium* hybrid groups. A wide spectrum of variation in symptom severity was observed in different isolate-genotype combinations. There was a good correlation between the lesion diameters on leaves and flowers of the *Lilium* genotypes, although the flowers generally showed faster expanding lesions. It was earlier postulated that *B. elliptica* pathogenicity on lily is conferred by secreted proteins that induce programmed cell death in lily cells. We selected two aggressive isolates and one mild isolate and collected culture filtrate (CF) samples to compare the cell death inducing activity of their secreted compounds in lily. After leaf infiltration of the CFs, variation was observed in cell death responses between the diverse lilies. The severity of cell death responses upon infiltration of the fungal CF observed among the diverse *Lilium* hybrid groups correlated well to their fire blight susceptibility. These results support the hypothesis that susceptibility to fire blight in lily is mediated by their sensitivity to *B. elliptica* effector proteins in a quantitative manner. Cell death-inducing proteins may provide an attractive tool to predict fire blight susceptibility in lily breeding programs.

## Introduction

Since the dawn of time, lily (*Lilium* spp., Liliaceae, L.) represents one of the most prestigious ornamental flowering plants in the Occidental culture as it was considered the symbol of divine purity. Either as bulb, potted plant or cut flower, lily is one of the main flower bulb crops sold worldwide (Kamenetsky, 2014). The genus *Lilium* consists of approximately 120 described species (Angiosperm Phylogeny Group (APG), 2020). Based on their geographical distribution and morphological traits (De Jong, 1974), ribosomal DNA sequences (Nishikawa et al., 1999; Nishikawa, 2007) and the chloroplast genome (Du et al., 2017; Kim et al., 2019), the genus *Lilium* is phylogenetically divided in seven taxonomic sections (Pelkonen and Pirttilä, 2012; Angiosperm Phylogeny Group (APG), 2020). The assortment of commercial lily genotypes includes thousands of hybrids that were generated via intrasectional crosses between different *Lilium* species and these have been divided into four hybrid groups: Asiatic (A), *Longiflorum* (L), Trumpet (T), and Oriental (O). To combine ornamental traits and obtain more vigorous plants, a variety of intersectional hybrids were subsequently generated using artificial pollination, embryo rescue and polyploidization techniques (van Tuyl et al., 1991; Lim et al., 2008; van Tuyl and Arens, 2011), resulting in the interspecific hybrid groups LA (*Longiflorum*-Asiatic), LO (*Longiflorum*-Oriental), OA (Oriental-Asiatic) and OT (Oriental-Trumpet). Despite its flourishing breeding and cultivation history, the lily industry is threatened by pests and diseases. Breeding efforts have thus far predominantly focused on ornamental traits and to lesser extent on the resistance to pests and pathogens. Given the current challenges more focus on disease resistance is needed.

Among several pests and diseases the most destructive is fire blight, a fungal disease caused by the filamentous Ascomycetes *Botrytis cinerea* and *Botrytis elliptica* (Doss et al., 1986; Fang Hsieh et al., 2001; Furukawa et al., 2005; Chastagner et al., 2017). Both fungi initially cause small brownish necrotic lesions on leaves and flowers. Outgrowth of necrotic lesions leads to rapid death of the entire plant. While the generalist *B. cinerea* was reported only to occur on damaged plant tissue and especially on cut lilies, the specialist *B. elliptica* inflicts serious economic losses since it is able to cause disease on undamaged, vigorous plants (Hou and Chen, 2003; Staats et al., 2007; Chastagner et al., 2017). The fungus can infect both leaves and flowers. Leaf infection can be devastating (hence the name “lily fire blight”), however this is usually controlled by intensive chemical control. Flower infection is a postharvest problem and is therefore economically more damaging, as it affects the quality at the retailer or leads to dissatisfied consumers. Both *B. cinerea* and *B. elliptica* belong to the genus *Botrytis* (Sclerotinaceae, Micheli ex Pers.) which includes around 35 described species (Valero-Jiménez et al., 2019). According to their necrotrophic lifestyle, *Botrytis* species kill the host cells to gain nutrients from the dead plant tissue for growth and reproduction (van Kan, 2006). The fungus is able to trigger the host plant cells to commit suicide via Programmed Cell Death (PCD) (van Kan, 2006; Veloso and van Kan, 2018). In the lily–*B*. *elliptica* interaction, PCD induction is mediated by secreted proteins, and the generalist *B. cinerea* and the tulip specialist *B. tulipae* acquired the ability to cause necrotic lesions when inoculated on lily leaves, previously infiltrated with *B. elliptica* secreted proteins (van Baarlen et al., 2004).

There are anecdotic reports among lily growers and breeders that certain lilies are less susceptible to *B. elliptica* than others. Published studies by Doss et al. (1986) reported a large variation in disease incidence (25–98%) and lesion size among 35 cultivars, of which the hybrid type was in most cases undescribed. A more recent study by Jang et al. (2018) also reported large variation in susceptibility among cultivars from four hybrid types and seven Korean genotypes from distinct indigenous species, with Asiatic hybrid genotypes generally falling in the highly resistant group, and the Korean genotypes generally falling in the susceptible group. A study by Gao et al. (2018), however, classified Oriental × Trumpet and Oriental hybrid cultivars as resistant, while Asiatic and Trumpet hybrids were classified as susceptible. Crosses between resistant and susceptible cultivars provided evidence for very complex inheritance of susceptibility to *B. elliptica* in hybrid progenies, with hybrid offspring displaying a large spectrum of susceptibility (Beers et al., 2005; Hu et al., 2017).

These previous studies, however, show several limitations that hamper obtaining a clear overview of the variation in susceptibility in lily and the variation in virulence in the *B. elliptica* population. Several studies included a single fungal isolate (Beers et al., 2005; Hu et al., 2017; Gao et al., 2018; Jang et al., 2018) and distinct inoculation methods. Sometimes agar plugs were used as inoculum (Hu et al., 2017; Gao et al., 2018), while in another case, leaf tips were inoculated with fungal conidia (Beers et al., 2005). Some studies were performed with cultivars of undescribed hybrid groups (Doss et al., 1986; Balode, 2009; Daughtrey and Bridgen, 2013) or performed in the field under unknown disease pressure and uncontrolled conditions (Balode, 2009; Daughtrey and Bridgen, 2013). Only a single study (Daughtrey and Bridgen, 2013) comprised repetitions over multiple years, and the method for disease scoring differed between all studies. Furthermore the variation in susceptibility of lily flowers was not addressed in any of the above mentioned studies.

We aimed to analyze and quantify the disease development under controlled conditions in a panel of commercial lily genotypes from seven hybrid groups upon inoculation with a collection of *B. elliptica* isolates. Disease assays were principally performed on leaves, and a subset of genotypes was also inoculated on flowers. We selected three fungal isolates that differed in virulence and evaluated the capacity of their culture filtrates (CFs) to cause cell death in leaves of different lily genotypes. These experiments enabled us to investigate a correlation between the susceptibility of lily genotypes to fire blight and their sensitivity to cell death induction in response to fungal secreted compounds.

## Materials and Methods

### Plant Material and Growth Conditions

Eighteen *Lilium* genotypes (Table 1) were used in this study representing the hybrid groups Asiatic (A), Longiflorum (L), Oriental (O) or intersectional hybrid groups Longiflorum × Asiatic (LA), Longiflorum × Oriental (LO), Oriental × Asiatic (OA), Oriental × Trumpet (OT), respectively. Material was provided by breeding companies as fresh bulbs and stored in potting soil at −1°C in darkness until planting. For commercial reasons, the names of cultivars have been anonymized. Lilies used in the disease assays in early spring (April–May 2019 and 2020) were planted as bulbs in the second week of February 2019 and 2020, whereas lily bulbs used in the disease assays in late spring (May–June 2019 and 2020) were planted in the third week of March 2019 and 2020. For repetitions of the assay, each of the 18 lily genotypes was planted in batches of 10 bulbs in plastic crates containing potting soil and grown in a greenhouse under natural day/night light regime at a minimum night temperature between 12 and 15°C and a maximum day temperature between 24 and 26°C. Temporal differences in shoot emergence from soil, vegetative growth and transition into flowering were observed. Disease assays in the early spring were conducted with mature leaves (as defined by Bar and Ori, 2014) of 6–7 weeks old plants in their vegetative stage, whereas disease assays in the late spring were conducted with mature leaves of 4–5 weeks old plants in their vegetative stage.

**TABLE 1:** Lily genotypes used in this study.

Lily hybrid group	Genotype		
Asiatic (A)	A 1^1,2,3^	A 2^1,3^	A 3^1,2^
Longiflorum (L)	L 1^1^	L 2^1,2,3^	L 3^1,2,3^
Oriental (O)	O 1^1,2,3^	O 2^1,3^	O 3^1,2^
Longiflorum × Asiatic (LA)	LA 1^1,2,3^	LA 2^1,3^	LA 3^1,2^
Longiflorum × Oriental (LO)	LO 1^1,2^	LO 2^1,2,3^	
Oriental × Asiatic (OA)	OA^1,3^		
Oriental × Trumpet (OT)	OT 1^1,3^	OT 2^1,2^	OT 3^1,2,3^

*^1^Lily genotypes used for disease assays in leaves. ^2^Lily genotypes used for disease assays in flowers and leaves. ^3^Lily genotypes used for leaf infiltration with culture filtrate samples.*

Disease assays on flowers were conducted in June 2020. Lilies used for disease assays on flowers were planted as bulbs in the second week of May 2020 and grown in a greenhouse under natural day/night light regime. Depending on the genotype, flower buds started to develop after 4–6 weeks. Lilies carrying flower buds at initiation of bud color were harvested as cut flowers and transported to the laboratory to be inoculated. Fungal inoculations on lily flowers were conducted within 2 days after bud opening. From the same lilies used for inoculation on flowers, leaves were detached to be inoculated with fungal conidia.

### Fungal Material and Growth Conditions

*Botrytis elliptica* isolates used in this research (Table 2) were stored as conidia suspensions in 20% glycerol at −80°C. To obtain conidia for inoculation, fungi were grown on Malt Extract Agar (50 g/L, Oxoid) at 20°C and sporulation was induced by illumination with UV-A lamps. After harvesting, the conidia were collected and washed in demineralized water, counted with the Bürker-Türk counting chamber, adjusted to a concentration of 10^6^ conidia/mL and stored until use in darkness at 4°C. Disease assays with *B. cinerea* were conducted similarly using isolate B05.10 (van Kan et al., 2017).

**TABLE 2:** *Botrytis* isolates used in this study.

Isolate code	Year of isolation	Sampling location
Be9174^1^	1991	Unknown
Be9401^1,2,3^	1994	Lisse (NL)
Be9605^1^	1996	Lisse (NL)
Be9610^1^	1996	Bergentheim (NL)
Be9612^1^	1996	Anerveen (NL)
Be9714^1^	1997	Elsloo (NL)
Be9732^1,2,3^	1997	Lisse (NL)
Be0006^1,2,3^	2000	Rutten (NL)
Be254-2^1^	Unknown	Unknown
Be254-8^1^	Unknown	Unknown
Be254-11^1^	Unknown	Unknown
Be748^1^	Unknown	Unknown
Bcin_B05.10^2^	1995	Münster (Germany)

*Be, Botrytis elliptica; Bc, Botrytis cinerea. ^1^Fungal isolates used for disease assays in leaves. ^2^Fungal isolates used for disease assays on flowers and leaves. ^3^Fungal isolates used for production of culture filtrate samples.*

### Disease Assays on Lily Leaves

Four rounds of disease assays were conducted in 2019 and 2020, both in the early (April–May) and late spring (May–June) season. In each round of assays all 18 lily genotypes were tested with 12 *B. elliptica* isolates. Nine lily genotypes were tested in two consecutive weeks, each week with six *B. elliptica* isolates, for a total of 4 weeks per round. Each disease assay was carried out as follows. From each genotype, 30 mature leaves (as defined by Bar and Ori, 2014) were detached from 10 plants and transported in clean plastic bags to the laboratory for inoculation. Per genotype, three random detached leaves were placed in plastic boxes on plastic grids placed on a layer of wet filter paper, with a total of three lily genotypes per box. The leaves were inoculated with each *B. elliptica* isolate at a concentration of 10^5^ conidia/mL in Potato Dextrose Broth (12 g/L, PDB, Oxoid). Leaves were abaxially inoculated with four 2 μL droplets of conidial suspension on well separated spots avoiding leaf edges and leaf veins. In total, 12 inoculations were performed per genotype and per isolate in each round of disease assay. Mock inoculation was carried out in separate boxes on three detached leaves with 2 μL droplets of PDB. Plastic boxes were closed with a transparent plastic lid and sealed with tape to maintain high humidity. After 4 days of incubation at constant temperature (19–21°C) and under regular day/night light regime, inoculated leaves were photographed and lesion diameters measured with a caliper. Given the ellipsoidal shape of necrotic spots, the lesion diameter was measured as a conjugate diameter^1^ (accessed January 2021). Specifically, we measured the length of the chord that connects the middle of the top-left quadrant with the middle of the bottom-right quadrant of the ellipse (illustrated with white dotted lines in Supplementary Figure 1A).

### Disease Assays on Lily Flowers

To compare the susceptibility to *B. elliptica* of flowers and leaves and to test the pathogenicity of *B. cinerea* on lily, combined disease assays on flower and leaf tissues were conducted in June 2020. In these experiments, leaves and flowers of 12 lily genotypes were used, representing all but one of the hybrid groups (Table 1). Conidia of three *B. elliptica* isolates that showed variation in aggressiveness in leaf assays (Table 2) and of *B. cinerea* isolate B05.10 were inoculated on flowers and leaves with 2 μL droplets of conidial suspension at a concentration of 5 × 10^4^ conidia/mL PDB (6 g/L). Disease assays on flowers were carried out with two just opened flowers that were harvested from the inflorescence of lilies and fixed into wet oasis foams, which were placed into plastic boxes containing tap water. Of the six tepals of each flower, five were inoculated with one droplet of conidial suspension and one was marked with paper tape and mock inoculated. Simultaneously to the flowers, three leaves per genotype were inoculated with the same fungal isolates. After 3 days of incubation at constant temperature (19–21°C) and under regular day/night light regime, the inoculated plant material was photographed and lesion diameters were measured.

### Production of *B. elliptica* Culture Filtrate and Leaf Infiltration

*Botrytis elliptica* CF were obtained by growing each isolate (Be9401, Be0006, and Be9732) in a 250 mL flask containing 50 mL of liquid medium with 3g/L Gamborg B5 salts (Duchefa, Haarlem, Netherlands), 10 mM potassium phosphate pH = 6 (LabChem, Tiel, Netherlands), 1% sucrose (Duchefa) and 30% lily leaf extract in demineralized water. The leaf extract was obtained by grinding with a blender 30 g of fresh harvested leaf material of genotypes A 3, L 2 and OT 3 (10 g each) in 250 mL demineralized water. The homogenate was centrifuged (3500 rpm, 20 min) and the supernatant was concentrated by freeze drying. The obtained concentrate was filter-sterilized (0.45 μm pore size, Millipore) and added to the liquid medium. The liquid culture was started at a spore concentration of 10^4^ conidia/mL medium. Flasks were closed with a cotton plug and fungal cultures were grown in a shaking incubator for 7 days (20°C, 150 rpm). When the liquid cultures were harvested, pH of the CF was adjusted to 6.0 with 1M KOH, and the CF was passed through a layer of Miracloth (Calbiochem, San Diego, CA, United States), filter-sterilized (0.45 μm pore size, Millipore, Amsterdam, Netherlands) and ice-chilled. A mock liquid medium was prepared and processed in the same way without fungal conidia added. For leaf infiltration with the CF samples and mock solution, three detached leaves were used of non-flowering plants from 12 different lily genotypes, representing all seven lily hybrid groups (Table 1). The leaves were placed in moist plastic boxes and 0.1 mL CF sample from each fungal culture was abaxially infiltrated in four different spots per leaf with the aid of a 1 mL sterile plastic syringe. After 3 days incubation at 19–21°C under ambient daylength, leaves were photographed and the response evaluated.

### Statistical Analysis

The statistical analysis was performed using R (Version 4.0.2). Lesion diameters measured in disease assays on lily leaves and flowers were plotted on a box-plot diagram using ggplot^2^ (Version 3.2.2). To determine the contributions of plant and pathogen genotype, and the seasonal effect on observed variation, the following linear mixed model was used:

$$\begin{array}{c} ls=be+group+be:group+season\\ +season:group+season:be+year{}_{R} \end{array}$$

whereby: *ls*, lesion diameter measured; *be*, *B. elliptica* isolate; *group*, lily hybrid group; *season*, late or early spring; *year*, 2019 or 2020, as random variable.

As the data were not balanced, a REML model was built using lmer() from the lme4 package (Version 1.1-22; Bates et al., 2015). The year was added to the model as a random effect to correct for year effect. To quantify the impact of the year on the variation observed, a variance component analysis was performed with lme4. Estimated marginal means (EMMs) of lesion diameters per fungal isolate, per hybrid group, were obtained using emmeans (Version 1.5.0). Differences in EMMs were calculated using the pairwise comparison function of emmeans, and *p*-values for differences in EMMS were adjusted by Tukey’s method. Pairwise differences for the average lesion diameters within isolate, for each hybrid group were plotted on a dot plot using ggplot().

Lesion diameters measured in lily flowers and in leaves of cut lilies were analyzed with the following linear model.

ls=be+group+group:be

whereby: *ls*, lesion diameter measured; *be*, *B. elliptica* isolate; *group*, lily hybrid group.

This simpler, linear model was chosen because the flower infection assays were performed only in one season (early summer 2020).

EMMs were obtained using emmeans (). Differences in EMMs were calculated and plotted similarly to the EMMs for lesion diameter on leaves.

## Results

To assess the susceptibility of lily genotypes to *B. elliptica* and the virulence of *B. elliptica* isolates in a quantitative manner, we inoculated 12 fungal isolates (Table 2) on leaves of 18 lily genotypes (Table 1) representing three hybrid groups (Asiatic, Longiflorum and Oriental) and four intersectional hybrid groups (LA, LO, OA, OT). Lesion development was quantified over the course of 3–4 days post inoculation (dpi) (raw data available in Supplementary Material 1). The experiments were conducted in four replicates over 2 years and each experiment contained 12 inoculation spots per genotype-isolate combination.

### Disease Assays in Leaves Distinguish Sections and Interspecific Hybrids

**FIGURE 1:**
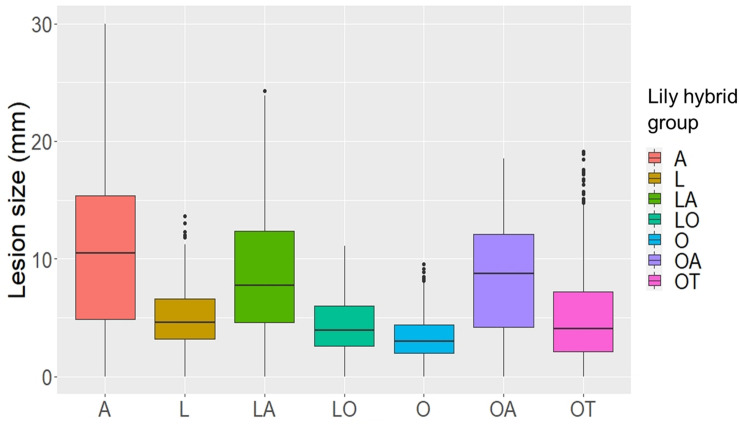
Lesion diameters at 4 dpi upon inoculation of 12 different *B. elliptica* isolates on leaves of seven different lily hybrid groups. Each box represents all compiled lesion diameters (in mm) for a given lily hybrid group in all four repetitions of the disease assays.

**FIGURE 2:**
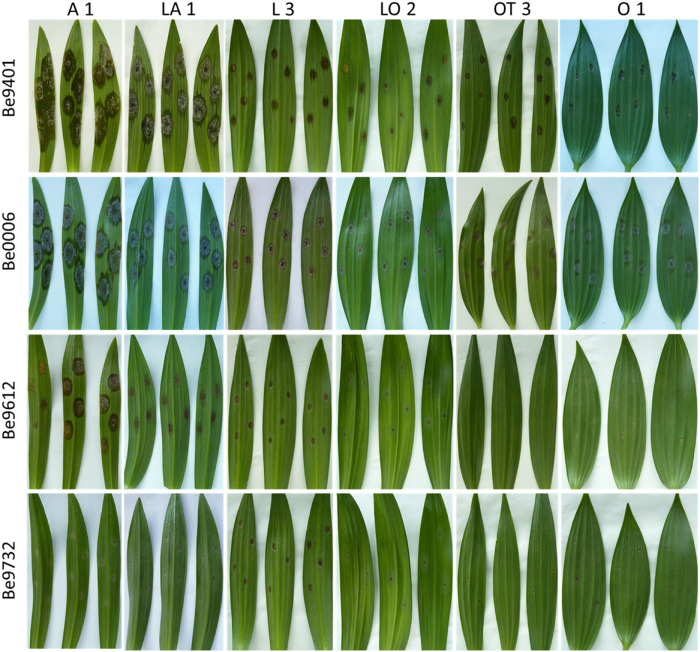
Necrotic lesions at 4 dpi upon inoculation with four distinct *B. elliptica* isolates (specified in left margin) on leaves of six lily genotypes (specified at the top). For each repetition of the disease assay, three representative leaves are shown.

Upon inoculation with conidial suspensions, disease symptoms initially appeared as small necrotic spots of brownish, collapsed abaxial epidermal tissue which were surrounded by a water soaked translucent area (Supplementary Figures 1, 2). In the most susceptible genotypes the lesions became visible from 2 dpi onward (Supplementary Figure 2). In the following days, the necrotic areas expanded, a larger portion of the abaxial leaf epidermis collapsed and the mesophyll tissue started to shrink inwards until the necrotic tissue could be observed also on the adaxial side of the leaf. With disease progression, the typical fire blight ellipsoidal necrotic lesions developed and especially for the most virulent isolates, growth of fungal mycelium was observed on the dead leaf tissue. Moreover, a dark discoloration was observed in the leaf vasculature and leaf yellowing was observed at a distance from the necrotic lesion. Mock inoculated leaves remained symptomless for the entire incubation period (Supplementary Figure 2). At 4 dpi, symptoms were quantified by measuring the lesion diameter. Despite the variation in lesion diameters among the specific genotype-isolate combinations, a general trend was noticed. Upon inoculation with all *B. elliptica* isolates, lily genotypes belonging to the groups A, LA, and OA developed larger lesions when compared to genotypes belonging to the groups L, O, LO, and OT (Figures 1, 2). As illustrated in Supplementary Figure 3, experiments in late spring (May–June 2019 and 2020) developed slightly larger lesions as compared to the experiments conducted in early spring (April–May 2019 and 2020). The largest variation in lesion diameter was observed among the groups A, LA, OA, and OT where lesion diameters ranged from 0 to 30 mm in the A genotypes and from 0 to 20 mm in the LA, OA and OT groups (Figure 1). By contrast, lily genotypes belonging to the groups L, O, and LO showed lesion diameters that ranged from 0 to 12 mm (Figure 1). This trend was consistently observed in both years and seasons (Supplementary Figure 3).

### Variation Between Genotypes Within Sections and Interspecific Hybrids

**FIGURE 3:**
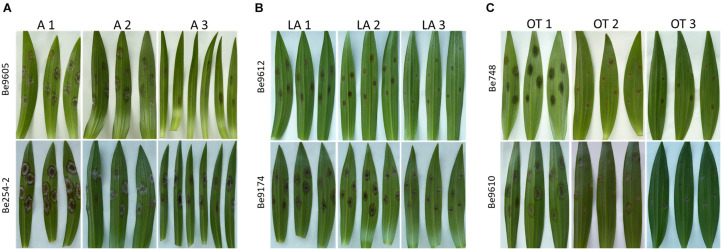
Necrotic lesions at 4 dpi on leaves of three different lily genotypes belonging to the hybrid group Asiatic inoculated with Be9605 and Be254-2 **(A)**, hybrid group LA inoculated with Be9612 and Be9174 **(B),** and hybrid group OT inoculated with Be748 and Be9610 **(C)**.

Differences in lesion diameters were not only detected between the different lily hybrid groups but also between individual genotypes within the same hybrid group (Figure 3). The most evident differences were observed when comparing lily genotypes of the groups A (Figure 3A), LA (Figure 3B), and OT (Figure 3C). In genotypes A1 and A2 the lesion diameters ranged from 15 to 25 mm, whereas in genotype A3 only the more aggressive fungal isolates caused lesions larger than 10 mm (Figure 3A). In the genotypes LA1 and LA2 lesion diameters ranged from 15 to 20 mm upon inoculation with the more aggressive isolates whereas in genotype LA3 the lesion diameters ranged from 10 to 15 mm (Figure 3B). In genotype OT1 lesion diameters ranged from 12 to 18 mm whereas in genotypes OT2 and OT3 lesion diameters ranged from 4 to 12 mm (Figure 3C).

### Variation in Virulence Between Fungal Isolates

**FIGURE 4:**
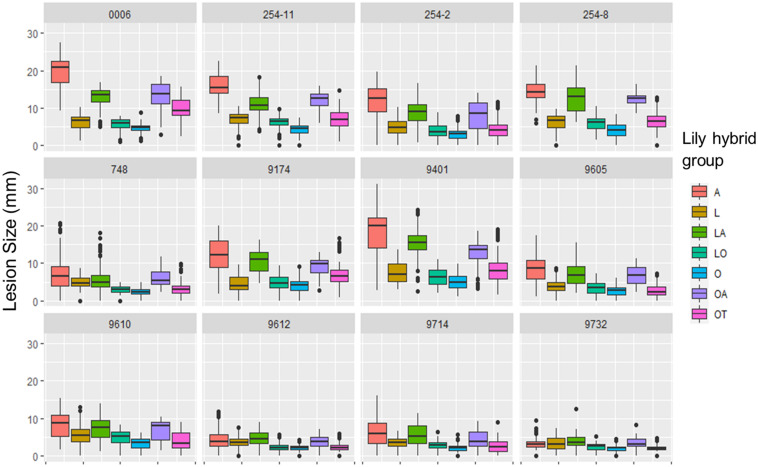
Lesion diameters at 4 dpi upon inoculation of 12 different *B. elliptica* isolates on leaves of 18 different lily genotypes. Lesion diameters measured in mm are plotted for each single isolate in 12 separate panels. Each of the colored boxes represents all lesion diameters measured for a given lily hybrid group in all four repetitions of the disease assays.

The variation in lesion diameter and symptom development was also related to differences in virulence of the tested *B. elliptica* isolates (Figures 2, 4). All 12 *B. elliptica* isolates were able to cause disease symptoms on leaves of all lily genotypes. Inoculation with Be9401 and Be0006 resulted in lesions ranging from 10 to 30 mm on leaves of susceptible hybrid groups A, LA, and OA, and ranging from 8 to 20 mm on OT genotypes. These two isolates were classified as aggressive. By contrast, inoculation with isolates Be9612, Be9714, and Be9732 resulted in lesions ranging from 3 to 12 mm in the susceptible hybrid groups A and LA, and even smaller lesions on less susceptible lily groups (Figure 4), classifying these three isolates as mild pathogens. The lesions diameters of the other seven isolates classified these as intermediate aggressive. All isolates, including the aggressive ones, developed lesions < 10 mm when inoculated on leaves of the groups O and LO and lesions < 15 mm on leaves of groups L and OT (Figure 4) confirming the relatively resistant nature of these lily hybrid groups.

### Statistical Analysis of the Variation in Lesion Diameter in the Disease Assays on Lily Leaves

**FIGURE 5:**
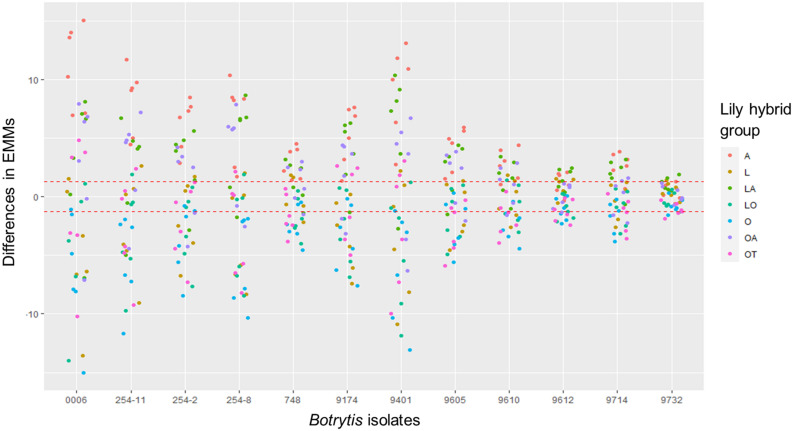
Dot plot showing differences in EMMs of lesion diameters on lily leaves in early spring for a given *B. elliptica* isolate, calculated using lily hybrid group as predicted factor. Red dotted line indicates the average *p*-value cut off for estimated differences which are significantly different from 0 (*p* > 0.05). *P*-values were adjusted using Tukey’s comparison for a family of 84 estimates.

The linear mixed model of lesion diameter on leaves shows that both the fungal diversity and lily group diversity, as well as the interaction between fungal isolate and lily group were the main contributors to the variation in lesion diameter (Table 3). The model shows that also the season and the interaction terms which included the season had a significant contribution on the variation in lesion diameter, while the year contributed to the variance only to a minimal extent (Table 3). Figure 5 and Supplementary Figure 4 show the differences in EMMs calculated for each lily hybrid group in the early and late spring, respectively (see also Supplementary Material 2). Each colored dot represents the estimated difference of the lesion diameter in one particular *B. elliptica* isolate – lily hybrid group interaction in comparison to the lesion diameter for the same *B. elliptica* isolate during the interaction with a different lily hybrid group. Three main patterns can be recognized:

(1) For all isolates in both seasons, dots representing the groups A (red), LA (green), and OA (violet) show a positive value in differences of EMMs. This indicates that in general, all isolates caused bigger lesions when inoculated on genotypes of these groups in comparison to genotypes of the groups L, O, LO, and OT. On the other hand, dots representing the groups O (light blue), LO (blue-green), and OT (pink) show negative values in differences of EMMs indicating that in general all isolates caused smaller lesions when inoculated on genotypes of these lily hybrids in comparison to genotypes of the groups A, LA, and OA. Finally, EMMs calculated for the interaction of each *B. elliptica* isolate with genotypes of group L (brown dots) cluster around the base line and in most of the cases they remain within the confidence intervals.
(2) For the more aggressive isolates (Be0006 and Be9401), larger differences in EMMs are observed when comparing the interaction of these isolates with the different hybrid groups. This indicates that the plant genotype significantly influenced the outcome of the isolate–plant interaction.
(3) The differences in EMMs for the isolates Be9612, Be9714, and Be9732 showed little variation, both within lily groups and between groups.

**TABLE 3:** ANOVA results of the interaction in leaves between 18 *Lilium* genotypes and 12 *B. elliptica* isolates measured as lesion diameter.

	Sum Sq	Mean Sq	NumDF	DenDF	*F* value	Pr(> F)
be	59305	5391.4	11	10272	960.3156	<2.2e-16***
group	74220	12370.1	6	10272	2203.3600	<2.2e-16***
season	7107	7106.6	1	10272	1265.8363	<2.2e-16***
be:group	26653	403.8	66	10272	71.9296	<2.2e-16***
group:season	247	41.2	6	10272	7.3365	7.62e-08***
be:season	1348	122.6	11	10272	21.8344	<2.2e-16***

**Random effects:**						

**Groups**	**Name (Intercept)**	**Variance**	**Standard deviation**			
Year		0.38747	0.62247			
Residual		5.61418	2.36943			
Number of obs: 10368, groups: year, 2						

*Type III analysis of variance with Satterthwaite’s method. P-value ≤ 0.05. Terms are as follows: be, the 12 B. elliptica isolates tested; group, the seven lily hybrid groups tested; season, early or late spring time period where the disease assays were conducted. In addition, interactions of these factors were tested (:). The degrees of freedom and p-values are shown. The experiment tests the random effect of four independent replicate experiments.*

### Disease Assays in Lily Flowers

The most appreciated organ in lily is the flower and fire blight is known to affect also the reproductive organs, especially at the cut flower stage (Munafo and Gianfagna, 2011). We examined the susceptibility of flowers among representatives of the seven lily hybrid groups. A pilot experiment revealed that the conidial density for flower inoculation needed to be reduced to prevent excessively rapid lesion outgrowth. The optimal density for discriminative infection assays on flowers was 5 × 10^4^/mL conidia, while for leaves we used a density of 10^5^/mL. For logistic reasons, the experiment could not be conducted with all 18 lily genotypes and 12 isolates. A panel of 12 lily genotypes (Table 1) was inoculated with three *B. elliptica* isolates (two aggressive isolates and one mild isolate; Table 2) and also one *B. cinerea* isolate and disease progression was monitored over time. *B. cinerea* was included because it was reported to cause necrotic lesions on lily flowers in the postharvest stage.

**FIGURE 6:**
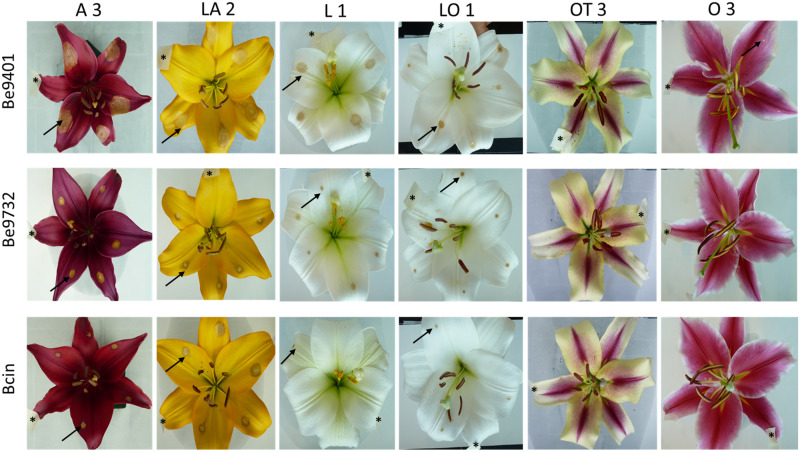
Necrotic lesions at 3 dpi upon inoculation of *B. elliptica* isolates Be9401, Be9732 and one *B. cinerea* isolate on flowers of lily genotype A 3, LA 2, L 1, LO 1, OT 3, and O 3. For each cultivar, one representative flower is shown that was inoculated on five different tepals. Black arrows indicate necrotic lesions observed on the tepals. Mock inoculation was labeled with a piece of paper tape on the tepal and is marked with a black asterisk (*).

**FIGURE 7:**
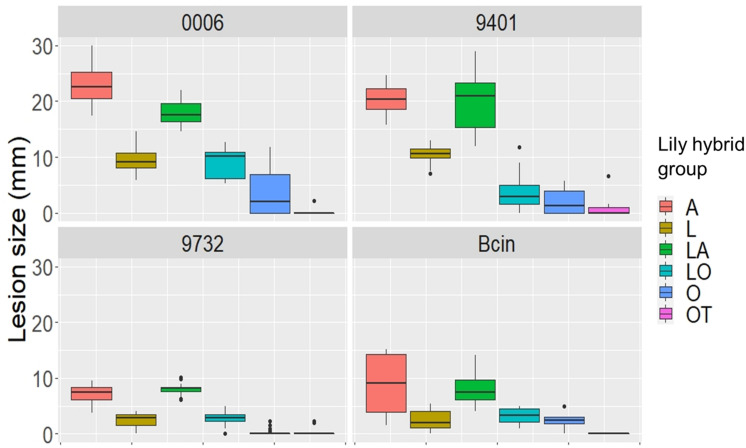
Lesion diameters at 3 dpi upon inoculation of *B. elliptica* isolates Be9401, Be9732, and Be0006 and one *B. cinerea* isolate on flowers of 12 lily genotypes. Lesion diameters measured in mm of each single fungus are plotted separately in the four panels. Each of the colored boxes represents all lesion diameters measured for a given lily hybrid group.

Because cutting of the inflorescence caused natural perigone abscission upon prolonged incubation, even in mock-treated samples and symptoms on lily flowers developed faster than on leaves and the size of necrotic lesions on tepals was therefore measured at 3 dpi (Table 4). All three *B. elliptica* isolates and *B. cinerea* were able to cause disease symptoms on lily flowers. As depicted in Figures 6, 7, flowers of lily genotypes belonging to the groups A and LA showed the most severe symptoms, irrespective of the isolate used. By contrast, lily genotypes of the groups O, LO, and OT showed smaller lesions upon inoculation with all four fungi (Figure 6, fifth and sixth column from the left). Inoculated flowers of L developed lesions with diameters that were in-between those observed on the groups A, O, LA, LO, and OT. Differences in lesion diameters were also observed among the fungal isolates when tested on flowers. *B. elliptica* isolates Be9401 and Be0006 caused larger lesions on flowers of all lily genotypes tested, as compared to Be9732 and *B. cinerea* (Figure 7 and Table 4).

**TABLE 4:**
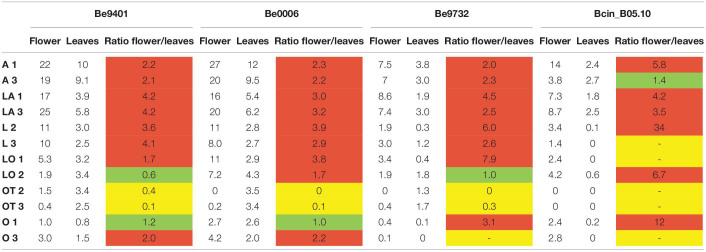
Ratios between average lesion diameters (given in mm) on lily flowers and leaves inoculated with three different *B. elliptica* isolates and one *B. cinerea* isolate at 3 dpi.

*Ratio < 0.5, flower less susceptible than leaf (yellow); 0.5 < ratio < 2, flower equally susceptible as leaf (green); ratio > 2, flower more susceptible than leaf (red).*

To compare *Botrytis* susceptibility of flowers and leaves as consistently as possible, we inoculated at the same spore density (5 × 10^4^/mL) conidia of the same three *B. elliptica* isolates and *B. cinerea* on leaves which were detached from the same lilies used to perform the flower assays. Overall, it was observed that at 3 dpi, inoculated leaves of lilies belonging to the groups A, L, LA, and LO developed smaller leaf lesions, as compared to flowers (Table 4). The trends in relative susceptibility between the hybrid groups and trends in differences in virulence among the isolates (Supplementary Figures 5, 6) were consistent with trends observed in the leaf inoculations on the entire set of lily genotypes with all fungal isolates (Figures 1, 2, 4). We calculated the ratios of lesion diameters on flowers and leaves from the same plants at 3 dpi and observed that for genotypes from the groups A, L, and LA, those ratios exceeded 1.5 for all three *B. elliptica* isolates, indicating that flowers were substantially more susceptible than leaves. For genotypes belonging to the hybrid group OT, the ratios were <0.5, indicating that leaves were more susceptible than flowers. For genotypes belonging to the hybrid groups O and LO, the ratios differed depending on the genotype-isolate combination (Table 4). Inoculation with *B. cinerea* conidia resulted in development of lesions of highly variable sizes on flowers of all hybrid groups, except OT (no lesions). By contrast, *B. cinerea* caused only small necrotic lesions on leaves of genotypes belonging to the groups A and LA, but rarely caused any lesions on genotypes of the other groups (Table 4 and Supplementary Figure 6).

The statistical analysis of the data for flower and leaf inoculations of cut lilies showed that the genetic diversity of fungal and plant genotype, and the interaction between fungal isolate–plant genotype significantly contributed to the variation observed in lesion diameter (Supplementary Tables 1, 2). When the differences in EMMs were calculated for the lesion diameters observed on flowers and on leaves of cut lilies (Supplementary Figures 7, 8), it was found that for all *Botrytis* isolates tested, dots representing the hybrid groups A (red dots) and LA (green dots) show a significant positive difference in EMMs. On the other hand, dots representing the groups O (light blue dots), LO (blue-green dots), and OT (pink dots) show negative differences in EMMs. The differences in EMMs calculated for the interaction of each *B. elliptica* isolate with L show a reduced spread and in several cases they remain within the confidence intervals. Moreover, it can be noticed that the differences in EMMs were larger when the interactions of the most aggressive isolates (Be0006 and Be9401) with a given lily genotype were compared, while smaller differences in EMMs were observed for the milder isolates (Be9732 and *B. cinerea*).

### Leaf Infiltration Assays With CF Samples

**FIGURE 8:**
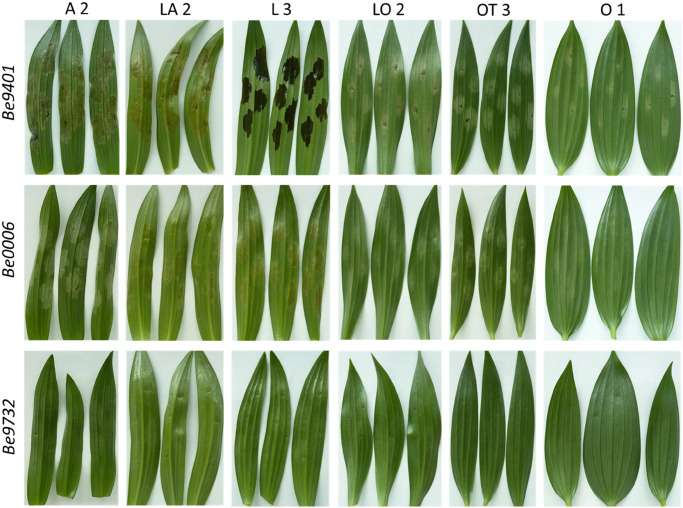
Cell death symptoms caused by infiltration of CF samples obtained from liquid cultures of three *B. elliptica* isolates (specified in left margin) on leaves of six lily genotypes (specified at the top).

Crude CF samples were collected from the same three *B. elliptica* isolates that were used in flower and leaf inoculations to test whether disease susceptibility and virulence correlate to effector sensitivity (of the lily genotype) and effector production (by the fungal isolate). CF samples were infiltrated in leaves of 12 lily genotypes (Table 1). At 3 days post infiltration, the leaf responses were observed (Figure 8 and Supplementary Figure 2). Lily genotypes displayed cell death responses of the infiltrated leaf area to different extents. A severe cell death response (tissue collapse, drying and slight browning) was observed for genotypes from the groups A, L, LA (Figure 8) and OA (Supplementary Figure 2) when infiltrated with CF samples from the aggressive Be9401 and Be0006. By contrast, a mild response (translucence, discoloration and softening of infiltrated tissue) was observed for genotypes representing the groups O, LO and OT when infiltrated with CF samples from these same isolates. The infiltration of genotype L3 with the CF from Be9401 resulted in symptoms very distinct from other combinations, typified by a dark discoloration, yet with a transparent appearance indicative of severe tissue degradation (Figure 8). The response of genotype L3 to CF samples from Be0006 and Be9732 was similar to that of many other genotypes tested. Moreover, CF from Be9401 did not cause similarly dark, macerated tissues in other lily genotypes. There were no visible responses in any lily genotype to infiltration with the CF sample obtained from Be9732 (Figure 8, third row). As expected, leaf infiltration with culture medium in which no fungi were grown did not induce any visible symptoms (Supplementary Figure 2).

## Discussion

### Variation in Fire Blight Susceptibility in *Lilium*

Our data unveil significant phenotypic variation in fire blight susceptibility within the phylogenetically complex genus *Lilium*. Genotypes belonging to the hybrid groups A, LA, and OA showed significantly more severe symptoms than genotypes of hybrid groups L, O, LO, and OT upon inoculation with all *B. elliptica* isolates tested (Figures 1, 2, 4, 5). This trend was consistently observed in all repetitions of the disease assays, both in leaves and flowers. These observations are in line with other studies, however our research provides more comprehensive data, as it included 18 lily genotypes, representing seven of the eight existing hybrid groups, as well as a set of 12 *B. elliptica* isolates collected over multiple years.

Asiatic (A) hybrids are obtained from lily species which belong to the taxonomic section *Sinomartagon* that is closely related to *L. longiflorum* (L). On the other hand, Trumpet (T) hybrids are generated from species which are closely related to species of the section *Archelirion* from which Oriental (O) hybrids derive (Nishikawa et al., 1999; Arzate-Fernández et al., 2005; Shahin et al., 2014; Kim et al., 2019). Intersectional hybrids LA, OA and OT were developed after chromosome doubling in the F1 progeny and backcrossing to A or O hybrids, respectively, in order to obtain a more vigorous, triploid genotype (van Tuyl and Arens, 2011). Breeding efforts have thus far predominantly focused on ornamental traits and to lesser extent on the resistance to pests and pathogens. Genetic loci associated with resistance to *Fusarium oxysporum* f. sp. *lilii* and lily mottle virus were mapped via QTL analysis (Arens et al., 2014). However, traits and loci related to *Botrytis* susceptibility remain unknown. This may be due to the fact that resistance to necrotrophic pathogens is often inherited as a recessive and quantitative trait (Wolpert and Lorang, 2016; Kariyawasam et al., 2016; Liu et al., 2017; Koladia et al., 2017; Faris et al., 2020) and may have not been considered during the breeding selection.

Although the A, LA, and OA genotypes tested were in general the more susceptible groups, there was substantial variation in lesion diameters and symptom severity between individual genotypes within these groups (Figures 3A,B). *Lilium* species of the section *Sinomartagon* (from which the Asiatic hybrids derive) are characterized by large genetic diversity because of their polyphyletic origin (Huang et al., 2018; Kim and Kim, 2018; Kim et al., 2019). Our results reveal that traits for reduced fire blight susceptibility (i.e., quantitative resistance) may be present in the germplasm of the Asiatic hybrids. Depending on the genotype(s) of the parents and of the line to which the F1 hybrid was backcrossed, traits conferring recessive resistance to fire blight may have been retained to different extents in the genomes of the new hybrid genotypes. It remains important to investigate a wider assortment of Asiatic hybrids for fire blight susceptibility. On the other hand, different genotypes of the hybrid group *Longiflorum*, showed little variation in lesion diameter upon inoculation with all *B. elliptica* isolates (Figures 1, 4). The genetic diversity of *Longiflorum* genotypes is narrower as compared to other sections, because either a single or very few species have been involved in the generation of these genotypes (van Tuyl, 1985).

### Flower Pathology in *Botrytis–Lilium* Interaction

*Botrytis* spp. are sometimes considered opportunistic pathogens which exploit wounded and senescing plant tissues to promote host infection. This assumption is supported by our results in the disease assays on flowers. Inoculated lily tepals developed larger necrotic lesions when compared to leaves of the same plant. Fungi of the genus *Botrytis* require PCD induction to infect plant tissue (van Kan, 2006; Veloso and van Kan, 2018) and during natural senescence of flower tissue, tepal cells begin to die before flower wilting becomes visible (van Doorn et al., 2003; van Doorn and Woltering, 2008; Rogers, 2013; Shibuya et al., 2016). In several monocot taxa (*Alstroemeria*, *Hemerocallis*, *Iris*, and *Lilium*) flower senescence shows typical hallmarks of PCD such as closure of plasmodesmata, starvation of tepal cells due to ATP depletion, formation of small vesicles, vacuolar swelling and tonoplast rupture. The progression of the PCD process involves the action of caspases and other intracellular proteases (Battelli et al., 2011), causing loss of cell integrity and breakdown of the cytoplasmic content including plastids and mitochondria, as well as the release of hydrolytic enzymes that degrade cell wall polysaccharides (van Doorn and Woltering, 2008; Rogers, 2013; Shibuya et al., 2016). Chromatin condensation and DNA fragmentation are considered as hallmark diagnostic features for PCD (van Baarlen et al., 2004; Yamada et al., 2006). Even though the PCD regulatory mechanisms at subcellular and molecular level are not fully understood, the micro-environment that is created during flower senescence (water-, sugar-, and amino acid-rich apoplast) represents an attractive growth substrate for necrotrophic pathogens. Therefore, the autonomous initiation and progression of PCD in cut flowers may benefit both *B. elliptica* and *B. cinerea* in their infection process and thereby cause more severe symptoms in lily flowers than in leaves.

The disease symptoms on lily flowers upon inoculation with *B. cinerea*, which is not specific to lily (van Baarlen et al., 2004), were comparable to those caused by the mild *B. elliptica* isolate Be9732. These observations suggest that these two fungi lack virulence factors that specifically promote infection in lily, however, they may benefit from the endogenous flower senescence process.

The relative ranking of flower susceptibility of lily hybrid types to fire blight showed a similar pattern to that in leaves. A and LA genotypes showed significantly larger lesions on flowers when compared to O, OT, and LO, with L genotypes showing intermediate lesion diameters. The ratio of lesion diameters in flowers versus leaves of the exact same plants (Table 4) showed a pattern that yet remains to be understood. The flowers of genotypes from the groups A, L, and LA were more susceptible than their leaves. By contrast, the leaves of genotypes from the OT group were more susceptible than their flowers, while genotypes from the groups O and LO showed a mixture of ratios, depending on the genotype-isolate combination. Whether *B. elliptica* uses different virulence factors for the infection of lily flowers and leaves will be subject of further studies.

### Fire Blight Susceptibility and Fungal Virulence Correlate to Effector Sensitivity and Effector Production

*Botrytis elliptica* secreted proteins can induce PCD when infiltrated in lily leaves (van Baarlen et al., 2004). We hypothesize that disease development in the *B. elliptica*-lily interaction is determined in a quantitative manner by the production by *B. elliptica* isolates of effector proteins that can induce PCD in the host, as well as by the sensitivity of lily genotypes to these effectors. Crude CF samples from cultures of *B. elliptica* were able to cause PCD in lily leaves to different extents. Genotypes belonging to the hybrid groups A, L, LA, and OA showed a more severe necrotic response upon CF infiltration, as compared to genotypes belonging to the groups O, LO, and OT (Figure 7 and Supplementary Figure 2F). These observations support the hypothesis that lily genotypes that developed the most severe disease symptoms in leaves and flowers were the same genotypes that showed a more pronounced necrotic response upon CF infiltration. Conversely, CF samples that caused the strongest necrotic response were obtained from *B. elliptica* isolates that caused the largest lesions on lily leaves and flowers in the disease assays, while CF from a less aggressive isolate caused little visible symptoms in any lily genotype.

The response observed in *Longiflorum* leaves upon infiltration with the CF sample from Be9401 was an outlier, despite the similar disease symptoms that this fungus caused upon inoculation. These leaves showed a much more severe response than all other infiltrated lily leaves. The infiltrated leaf segment had a dark brown color and yet appeared transparent, as if the tissue architecture was completely destroyed and the cell content dissolved. The dark pigmentation may have been generated by the combined activity of fungal and plant (poly)phenol oxidases which caused the oxidation of phenolic compounds released from dying lily cells during PCD (Schouten et al., 2002; Boeckx et al., 2015). At the same time, the leaf tissue collapse likely resulted from the action of fungal cell wall degrading enzymes in the CF sample, which cause maceration leading to loss of cell integrity and tissue architecture (van Kan, 2006). It is also important to consider that, differently from the situation in the disease assays, where necrotic lesions expand gradually over time upon fungal colonization, leaf infiltration with CF samples leads to the immediate and homogeneous exposure of all leaf cells to the PCD inducing compounds. Thereby, the activity of such compounds might become more effective and cause a stronger and faster response in the infiltrated leaf tissue. The physiological processes underlying the distinct response of the *Longiflorum* genotype could be unraveled by comparing the protein composition of CF samples from Be9401 with those of Be0006 and Be9732, the latter two causing a milder response in *Longiflorum* leaves (Figure 8, second row).

The broad spectrum of susceptibility (in the lily genotypes) and virulence (in the fungal isolates) suggests that there might be multiple effectors, each of them contributing quantitatively to PCD induction. In the *Parastagonospora nodorum*-wheat interaction, seven distinct cell death inducing effectors have been reported, each requiring a specific plant susceptibility gene (Friesen et al., 2008; Friesen and Faris, 2010; Zhang et al., 2011; Friesen et al., 2012; Gao et al., 2015; Shi et al., 2015). Three of these susceptibility genes, *Snn1* (Shi et al., 2016) and *Tsn1* (Faris et al., 2010) and *Snn3* (Zhang et al., 2021), have been cloned from wheat and appeared to encode proteins from distinct families of plant receptor genes that also contain many resistance genes. Recent work (Downie et al., 2018) has identified several additional QTLs in wheat germplasm that might also be involved in effector recognition to mediate susceptibility.

We hypothesize that the lily-*B*. *elliptica* interaction relies on similar effector-receptor interactions. At this point it is unknown how many effector proteins and their cognate receptors are at play in this plant–fungus interaction. The difference in susceptibility of the lily genotypes may be related to variation in the numbers and types of functional receptor proteins. In view of the huge genome size of lily, and the hybrid (diploid or triploid) nature of most genotypes, unraveling the molecular nature of such receptor genes will require a major effort. The *B. elliptica* effector proteins may be useful tools for mapping sensitivity genes and identify molecular markers for breeding purposes.

The difference in virulence between fungal isolates may be related to the repertoire of active PCD-inducing effector proteins that they secrete. Whether the most virulent isolates produce a different spectrum of effector proteins or possess more active allelic variants, or they produce larger quantities of effector proteins, remains to be analyzed once such effector genes are identified. Our next step will be to use mass spectrometry and identify proteins in the crude CFs that were generated in this study. The annotated genomes of two *B. elliptica* isolates (Valero-Jiménez et al., 2019, 2020) are instrumental in the identification of effector genes.

### Perspectives for Lily Breeding

We propose that the sensitivity of lily genotypes to *B. elliptica* effector proteins that induce PCD is predictive of the susceptibility of that individual plant to a fungal isolate that produces such effector(s). Given the logistic and experimental challenges of performing disease assays with living pathogens in a commercial breeding setting, PCD inducing proteins might provide an attractive alternative as prediction tool for fire blight susceptibility. Testing breeding material by a simple infiltration (of pure proteins or mixtures) and monitoring cell death responses presents a fast, low-tech solution to identify the most sensitive genotypes and eliminate them from the breeding program in early stages. This offers opportunities to pursue a breeding program only with a subset of breeding lines that will display increased fire blight resistance.

Such an “effectoromics” assay has multiple advantages. First of all, screening a population of young lily plants by protein infiltration represents a non-destructive assay since the infiltrated plant survives, and there is no (natural or artificial) infection of the fungus in the precious breeding population. Effector-sensitive genotypes can be discarded in a pre-breeding stage thereby saving time and costs. Multiple repeats on different organs of the same plant can be performed to obtain a more consistent scoring for effector sensitivity. Several thousand individual plants can readily be tested in 1 day. Furthermore, the good correlation that was observed between leaf and flower susceptibility is an encouraging incentive to presume that leaf infiltration of effector proteins is also predictive for fire blight susceptibility in flower. After a cross it usually takes 2 years before new offspring plants will be able to flower and testing leaves as a proxy for flower susceptibility gains precious time and saves greenhouse space and labor enabling to extend breeding efforts.

In other pathosystems, purified effectors have been implemented as tools to screen plant germplasm to predict disease resistance traits (Wolpert et al., 2002; Faris et al., 2010; Tan et al., 2012) and make recommendations to breeders. The development of a similar procedure to identify and eliminate the fire blight susceptible genotypes in a lily breeding population will support breeders in their efforts to develop new genotypes that may thrive with lower input of fungicide applications.

## Data Availability Statement

The original contributions presented in the study are included in the article/Supplementary Material, further inquiries can be directed to the corresponding author/s.

## Author Contributions

JK, PA, and MM designed the study. MM and TQ conducted the experiments and statistical analyses. MM wrote the draft manuscript. JK, PA, and RI edited the final manuscript. JK, PA, and RI supervised the study. All authors contributed to the article and approved the submitted version.

## Conflict of Interest

Plant material was provided by lily breeding companies. For reasons of competitiveness and publicity, the companies have requested that the names of cultivars would be anonymized in the manuscript. Other than this request, companies have neither had any influence on the research design nor on the interpretation of results, nor have they been involved in writing the manuscript.

## Footnotes

1 https://en.wikipedia.org/wiki/Conjugate_diameters

2 https://ggplot2.tidyverse.org/

## Supplementary Material

Supplementary Figure 1 **(A)** Fire blight symptoms observed upon and conidia inoculation of *B. elliptica* isolates *Be9605* on leaf of lily cultivar OT-1 at 3 dpi. White dotted lines represent the chord of the ellipsoidal necrotic spots used to measure the lesion diameter. **(B)** Close-up of two representative necrotic lesions highlighted in the black quadrant in **(A)**. Yellow arrows indicate area showing translucence, maceration softening and water soaking. Red arrows indicate necrotic collapsed tissue.

Supplementary Figure 2 Symptoms observed upon mock (left) and conidia inoculation of *B. elliptica* isolates Be9401 and Be9732 at 2 **(A)**, 3 **(B)**, and 4 dpi **(C)** on leaves of lily cultivar OA. Red arrows indicate translucence and maceration around the necrotic spot. Black arrows indicate dark brown coloration in the leaf vasculature. Cell death response observed at 3 days post infiltration of mock liquid medium **(D)**, Be9732 CF sample **(E)** and Be9401 CF sample **(F)** in leaves of lily genotype OA.

Supplementary Figure 3 Lesion diameters at 4 dpi upon inoculation of 12 different *B. elliptica* isolates on leaves of 18 different lily genotypes in early spring 2019 and 2020 **(A)** and in late spring 2019 and 2020 **(B)**. Each box represents all compiled lesion diameters measured in mm for a given lily hybrid group in the two seasonal repetitions of the disease assays.

Supplementary Figure 4 Dot plot showing differences in EMMs of lesion diameters on lily leaves in late spring for a given *B. elliptica* isolate, calculated using lily hybrid group as predicted factor. Red dotted line indicates the average *p*-value cut off for estimated differences which are significantly different from 0 (*p* > 0.05). *P*-values were adjusted using Tukey’s comparison for a family of 84 estimates.

Supplementary Figure 5 Lesion diameters at 3 dpi upon inoculation of *B. elliptica* isolates Be9401, Be9732, and Be0006 and *B. cinerea* isolate B05.10 on detached leaves of 12 cut lily genotypes. Lesion diameters measured in mm of each single fungus are plotted separately in the four panels. Each of the colored boxes represents all lesion diameters measured for a given lily hybrid group.

Supplementary Figure 6 Necrotic lesions developing at 3 dpi upon inoculation of Be9401, Be9732 and Bcin on leaves of cut lily genotypes A 3, LA 2, L 1, LO 1, OT 3, and O 3 from which also the flowers were tested. For each cultivar, 12 inoculations were performed on two or three leaves detached from fresh harvested lilies.

Supplementary Figure 7 Dot plot showing differences in EMMs of lesion diameters on lily flowers for a given *Botrytis* isolate calculated using lily hybrid group as predicted factor. Red dotted line indicates the average *p*-value cut off for estimated differences which are significantly different from 0 (*p* > 0.05). *P*-values were adjusted using Tukey’s comparison for a family of 24 estimated.

Supplementary Figure 8 Dot plot showing differences in EMMs of lesion diameters measured on leaves of lilies for a given *Botrytis* isolate calculated using lily hybrid group as predicted factor. Red dotted line indicates the average *p*-value cut off for estimated differences which are significantly different from 0 (*p* > 0.05). *P*-values were adjusted using Tukey’s comparison for a family of 24 estimated.

Supplementary Table 1 ANOVA results of the interaction in flowers between 12 *Lilium* genotypes and three *B. elliptica* and one *B. cinerea* isolates measured as lesion diameter. Analysis of Variance with Satterthwaite’s method. *P*-value ≤ 0.05. The experiment tests the random effect of one experiment. Terms are as follow: be, the three *B. elliptica* and one *B. cinerea* isolates tested; group, the six lily hybrid groups tested. In addition, interactions of these factors were tested (:). The degrees of freedom and *p*-value are shown.

Supplementary Table 2 ANOVA results of the interaction in leaves of cut lilies between 12 *Lilium* genotypes and three *B. elliptica* and one *B. cinerea* isolates measured as lesion diameter. Analysis of Variance with Satterthwaite’s method. *P*-value ≤ 0.05. The experiment tests the random effect of one experiment. Terms are as follow: be, the three *B. elliptica* and one *B. cinerea* isolates tested; group, the six lily hybrid groups tested. In addition, interactions of these factors were tested (:). The degrees of freedom and *p*-value are shown.

Supplementary Material 1 Lesion diameters measured at 4 dpi on 18 leaves of different lily genotype inoculated with 12 different *B. elliptica* isolates scored in the disease assays conducted in early (April–May) and late (May–June) spring 2019 and 2020. Lesion diameters measured at 3 dpi on flowers and leaves of 12 cut lilies genotypes inoculated with three *B. elliptica* isolates (Be9401, Be0006, and Be9732) and one *B. cinerea* isolate Bcin_B05.10.

Supplementary Material 2 Results of Multiple Comparison Analysis and EEMs conducted on lesion diameters scored in the four repetition of the main disease assays in lily leaves and on lesion diameters scored in the disease assays on flowers and leaves of cut lilies.
