# Supplementary figures and images for: Fire Blight Susceptibility in Lilium spp. Correlates to Sensitivity to Botrytis elliptica Secreted Cell Death Inducing Compounds
Source: Front Plant Sci. 2021 Jun 28;12:660337. doi: 10.3389/fpls.2021.660337 (PMC8273286; doi:10.3389/fpls.2021.660337)

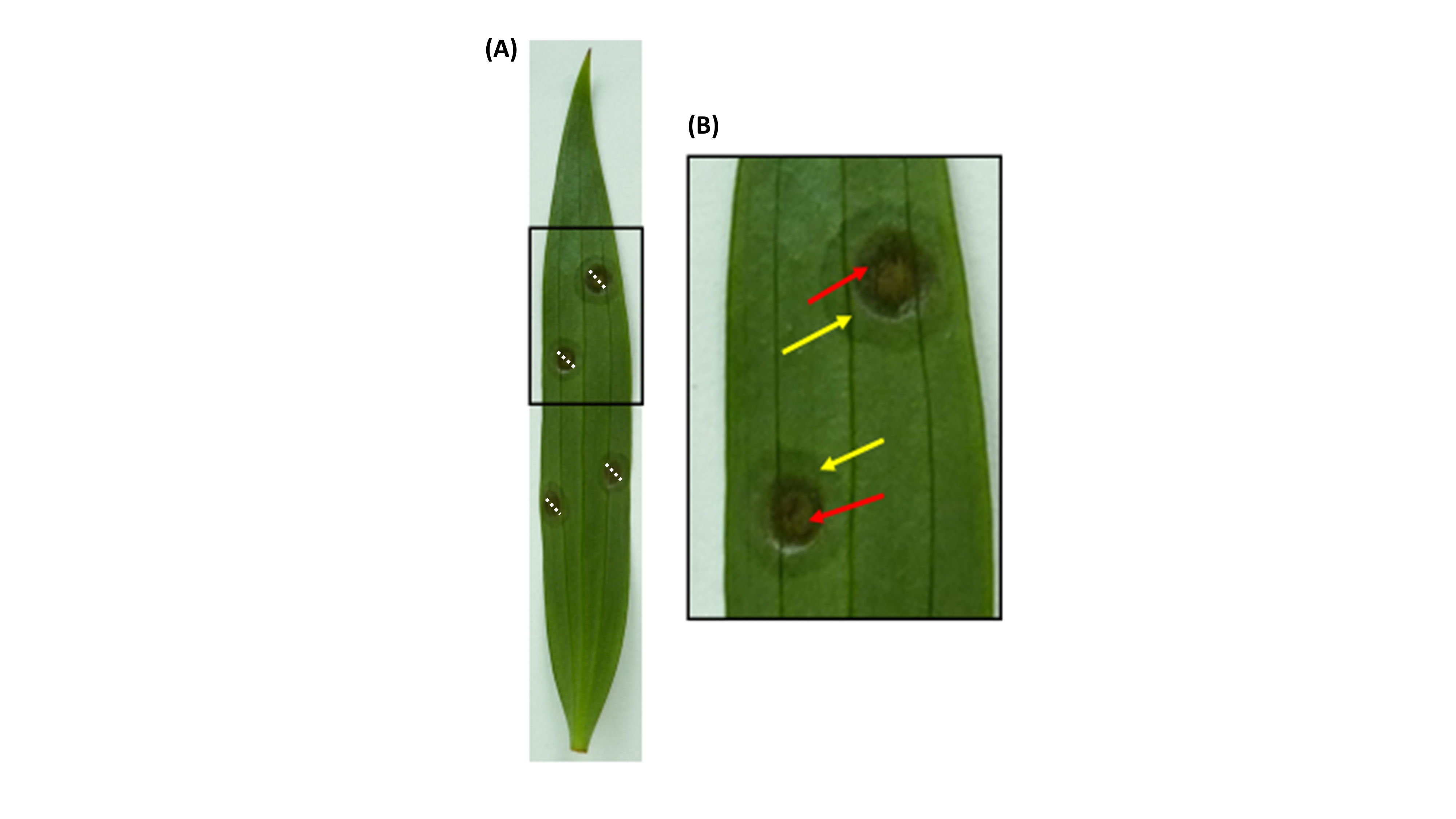

Supplement: Supplementary Figure 1 — (A) Fire blight symptoms observed upon and conidia inoculation of B. elliptica isolates Be9605 on leaf of lily cultivar OT-1 at 3 dpi. White dotted lines represent the chord of the ellipsoidal necrotic spots used to measure the lesion diameter. (B) Close-up of two representative necrotic lesions highlighted in the black quadrant in (A). Yellow arrows indicate area showing translucence, maceration softening and water soaking. Red arrows indicate necrotic collapsed tissue. [file Data_Sheet_1.zip › Supplementary Figure S1.TIF]

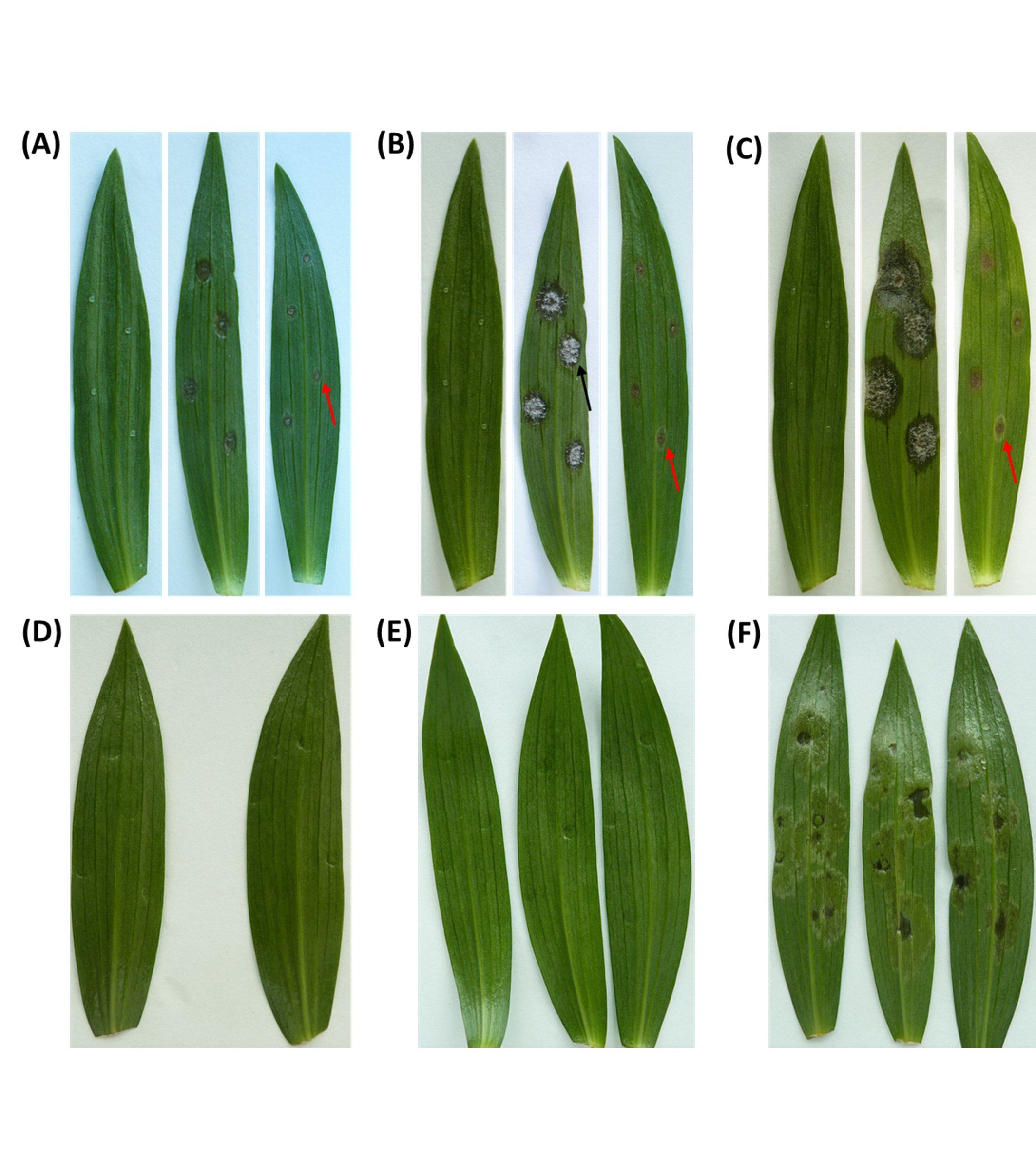

Supplement: Supplementary Figure 1 — (A) Fire blight symptoms observed upon and conidia inoculation of B. elliptica isolates Be9605 on leaf of lily cultivar OT-1 at 3 dpi. White dotted lines represent the chord of the ellipsoidal necrotic spots used to measure the lesion diameter. (B) Close-up of two representative necrotic lesions highlighted in the black quadrant in (A). Yellow arrows indicate area showing translucence, maceration softening and water soaking. Red arrows indicate necrotic collapsed tissue. [file Data_Sheet_1.zip › Supplementary Figure S2.TIF]

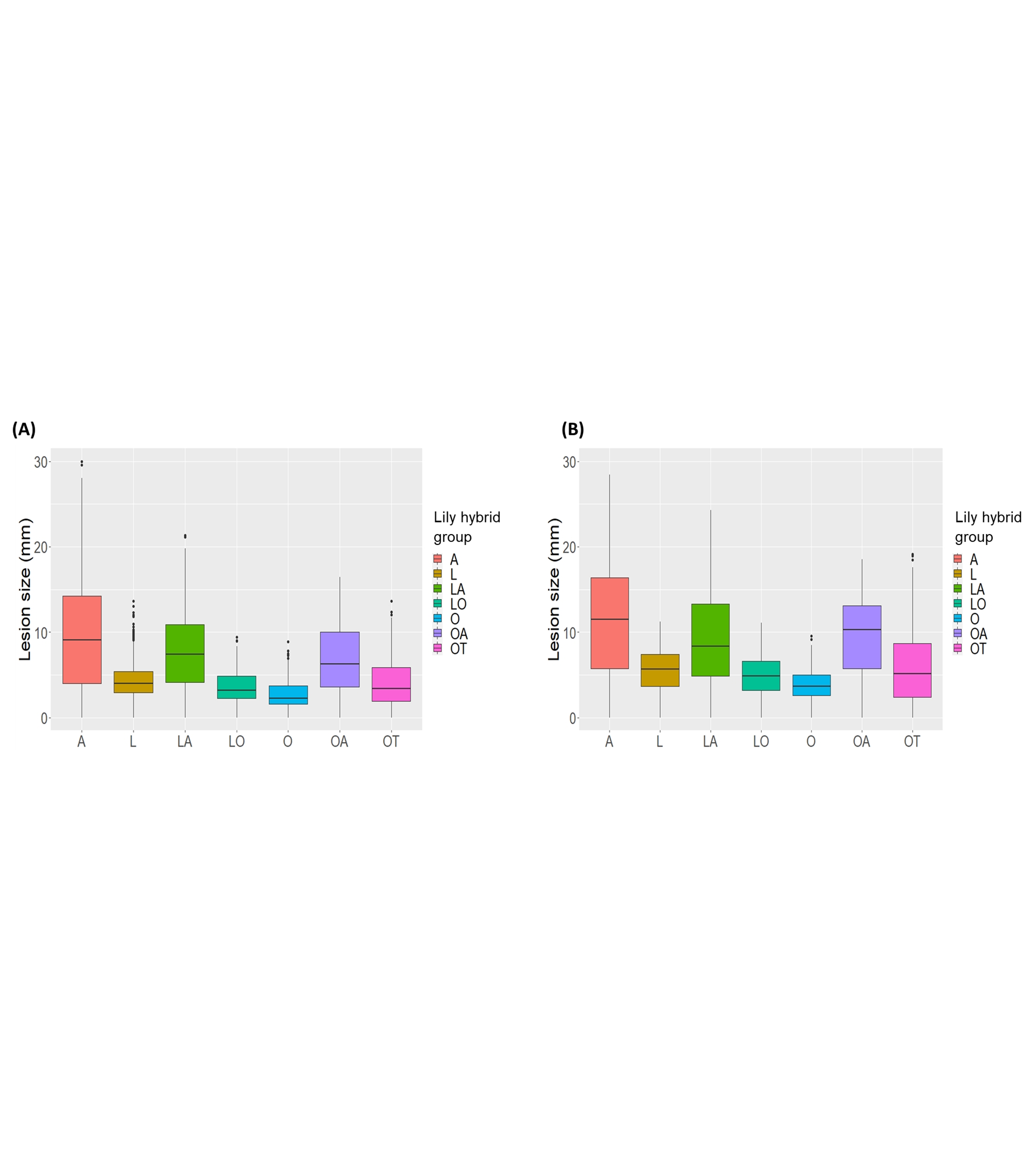

Supplement: Supplementary Figure 1 — (A) Fire blight symptoms observed upon and conidia inoculation of B. elliptica isolates Be9605 on leaf of lily cultivar OT-1 at 3 dpi. White dotted lines represent the chord of the ellipsoidal necrotic spots used to measure the lesion diameter. (B) Close-up of two representative necrotic lesions highlighted in the black quadrant in (A). Yellow arrows indicate area showing translucence, maceration softening and water soaking. Red arrows indicate necrotic collapsed tissue. [file Data_Sheet_1.zip › Supplementary Figure S3.TIF]

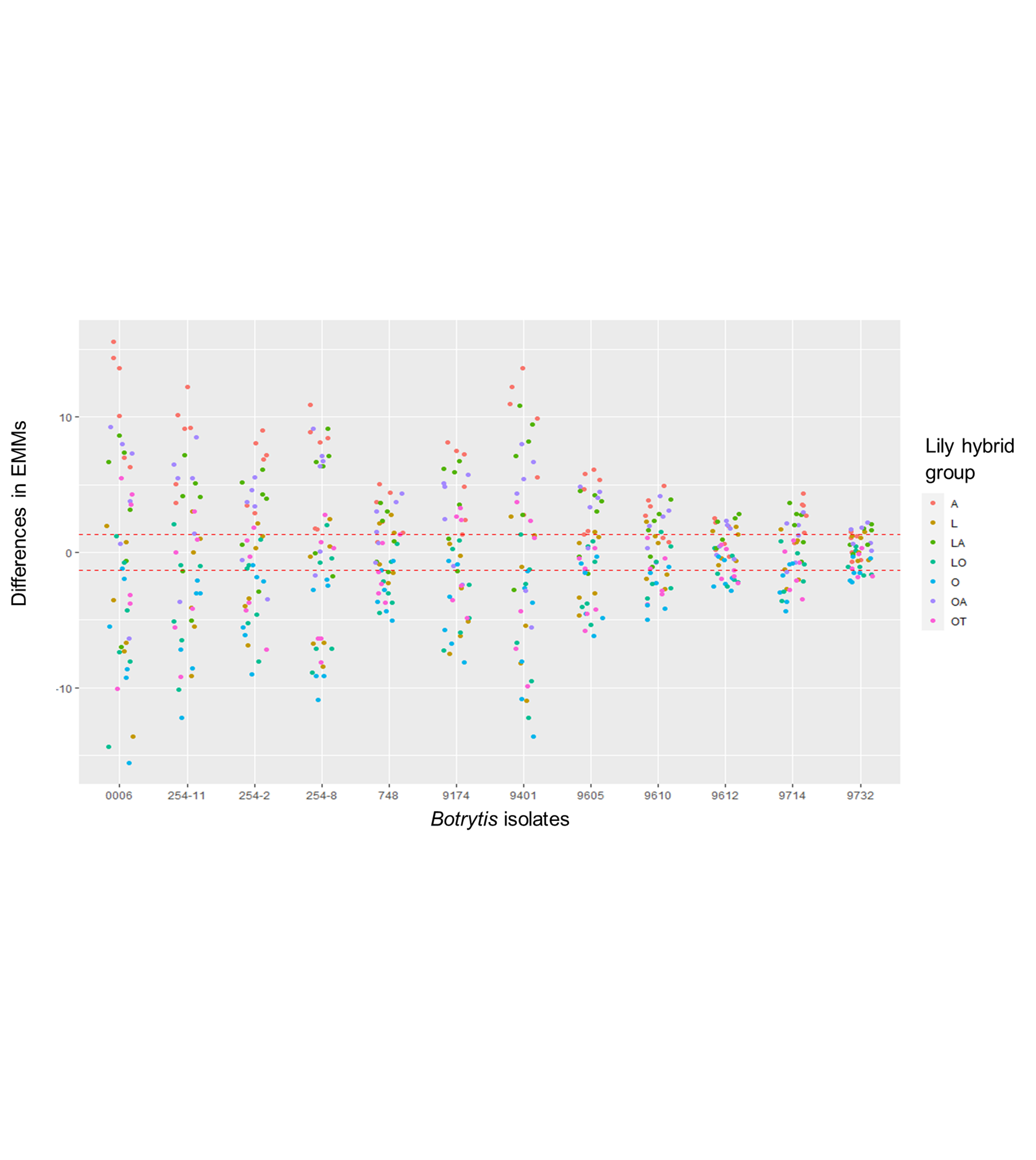

Supplement: Supplementary Figure 1 — (A) Fire blight symptoms observed upon and conidia inoculation of B. elliptica isolates Be9605 on leaf of lily cultivar OT-1 at 3 dpi. White dotted lines represent the chord of the ellipsoidal necrotic spots used to measure the lesion diameter. (B) Close-up of two representative necrotic lesions highlighted in the black quadrant in (A). Yellow arrows indicate area showing translucence, maceration softening and water soaking. Red arrows indicate necrotic collapsed tissue. [file Data_Sheet_1.zip › Supplementary Figure S4.TIF]

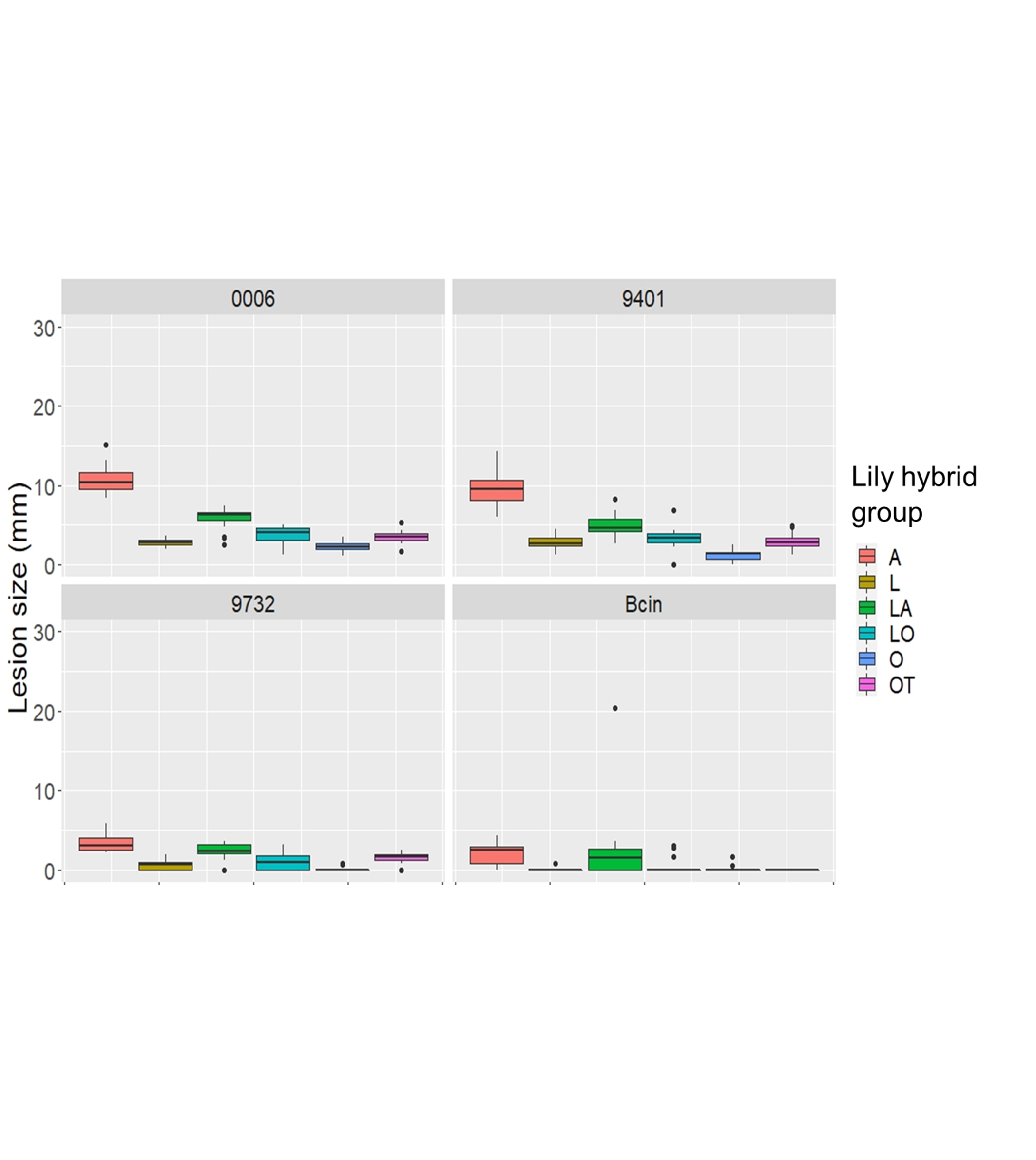

Supplement: Supplementary Figure 1 — (A) Fire blight symptoms observed upon and conidia inoculation of B. elliptica isolates Be9605 on leaf of lily cultivar OT-1 at 3 dpi. White dotted lines represent the chord of the ellipsoidal necrotic spots used to measure the lesion diameter. (B) Close-up of two representative necrotic lesions highlighted in the black quadrant in (A). Yellow arrows indicate area showing translucence, maceration softening and water soaking. Red arrows indicate necrotic collapsed tissue. [file Data_Sheet_1.zip › Supplementary Figure S5.TIF]

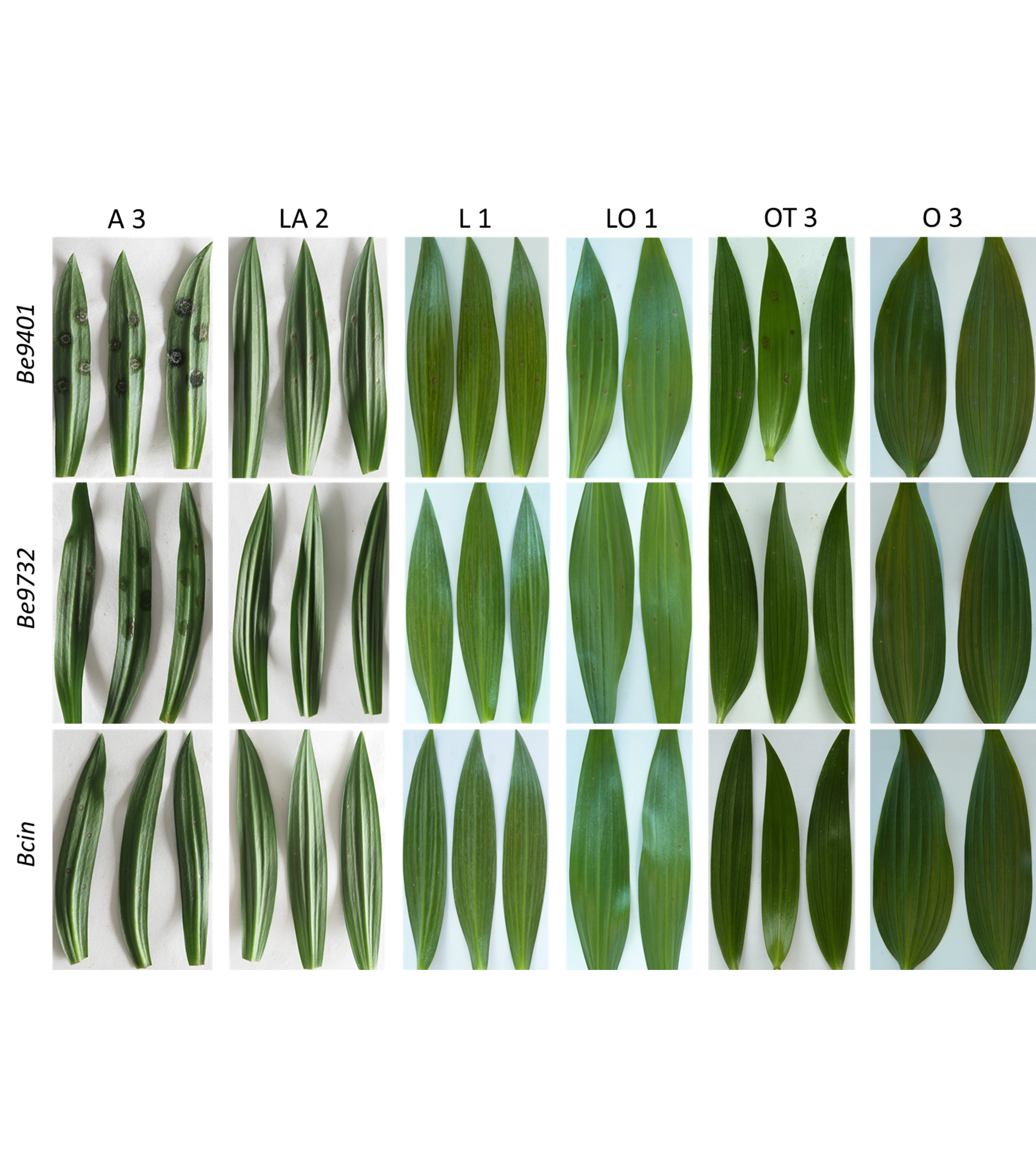

Supplement: Supplementary Figure 1 — (A) Fire blight symptoms observed upon and conidia inoculation of B. elliptica isolates Be9605 on leaf of lily cultivar OT-1 at 3 dpi. White dotted lines represent the chord of the ellipsoidal necrotic spots used to measure the lesion diameter. (B) Close-up of two representative necrotic lesions highlighted in the black quadrant in (A). Yellow arrows indicate area showing translucence, maceration softening and water soaking. Red arrows indicate necrotic collapsed tissue. [file Data_Sheet_1.zip › Supplementary Figure S6.TIF]

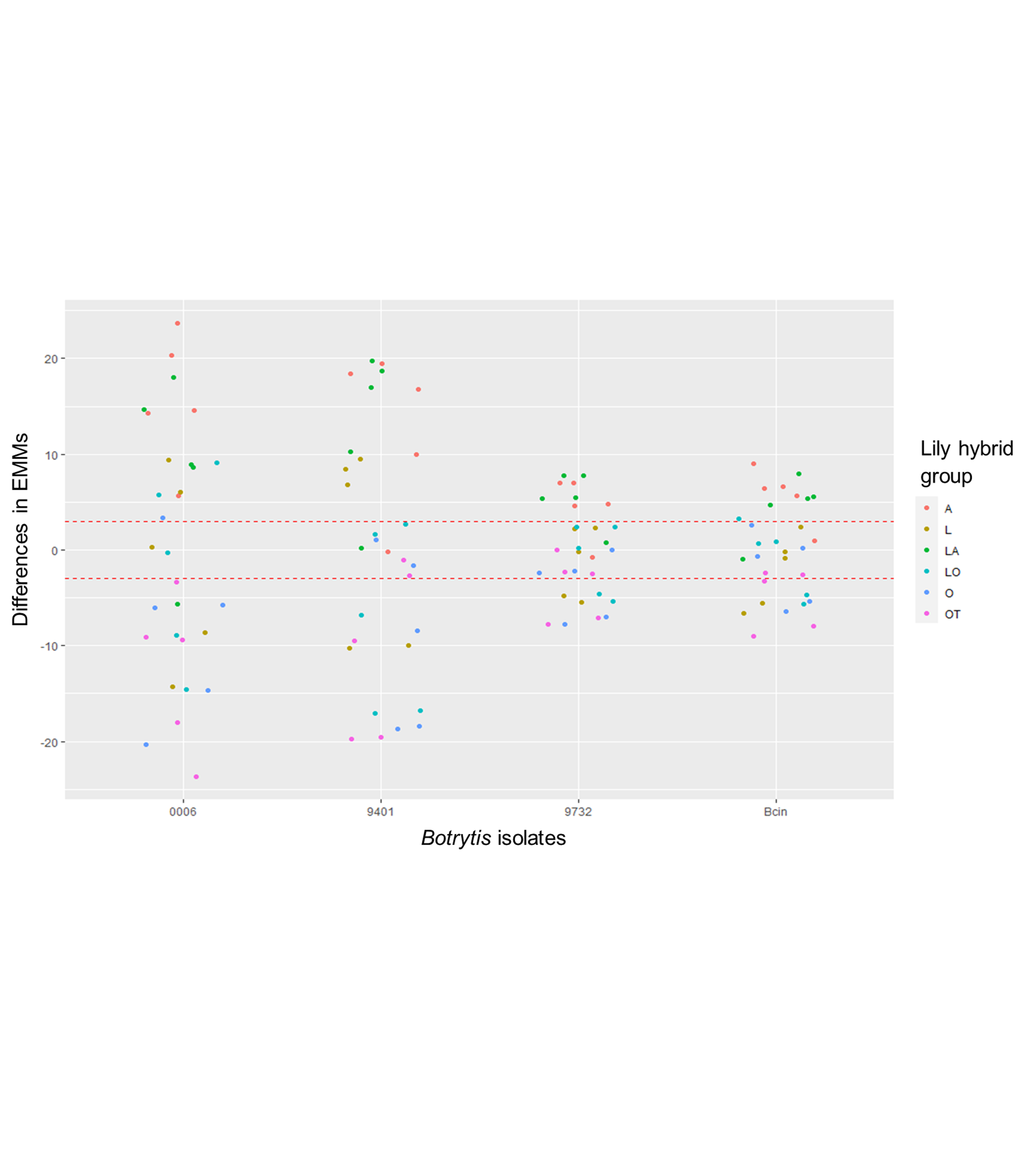

Supplement: Supplementary Figure 1 — (A) Fire blight symptoms observed upon and conidia inoculation of B. elliptica isolates Be9605 on leaf of lily cultivar OT-1 at 3 dpi. White dotted lines represent the chord of the ellipsoidal necrotic spots used to measure the lesion diameter. (B) Close-up of two representative necrotic lesions highlighted in the black quadrant in (A). Yellow arrows indicate area showing translucence, maceration softening and water soaking. Red arrows indicate necrotic collapsed tissue. [file Data_Sheet_1.zip › Supplementary Figure S7.TIF]

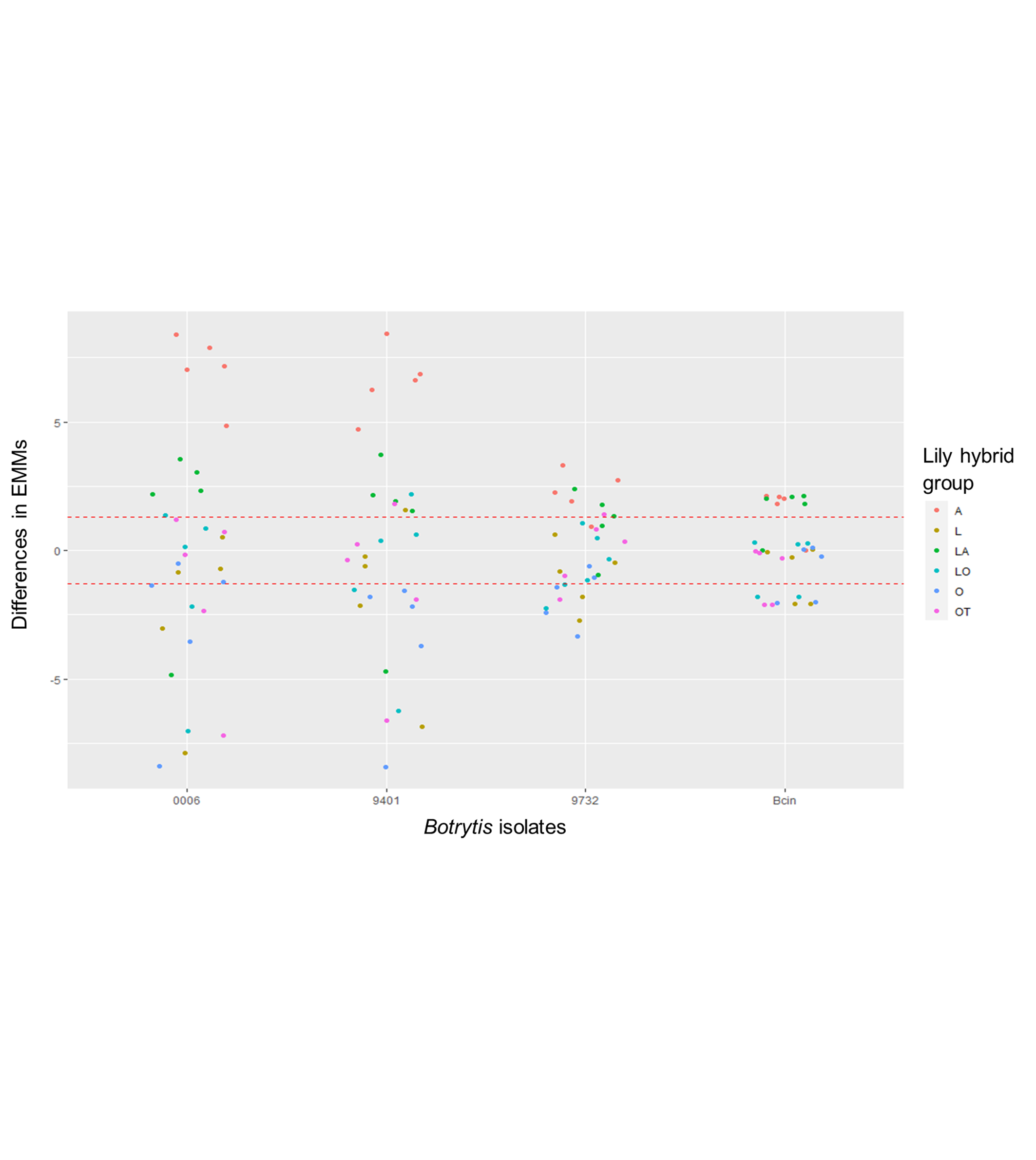

Supplement: Supplementary Figure 1 — (A) Fire blight symptoms observed upon and conidia inoculation of B. elliptica isolates Be9605 on leaf of lily cultivar OT-1 at 3 dpi. White dotted lines represent the chord of the ellipsoidal necrotic spots used to measure the lesion diameter. (B) Close-up of two representative necrotic lesions highlighted in the black quadrant in (A). Yellow arrows indicate area showing translucence, maceration softening and water soaking. Red arrows indicate necrotic collapsed tissue. [file Data_Sheet_1.zip › Supplementary Figure S8.TIF]

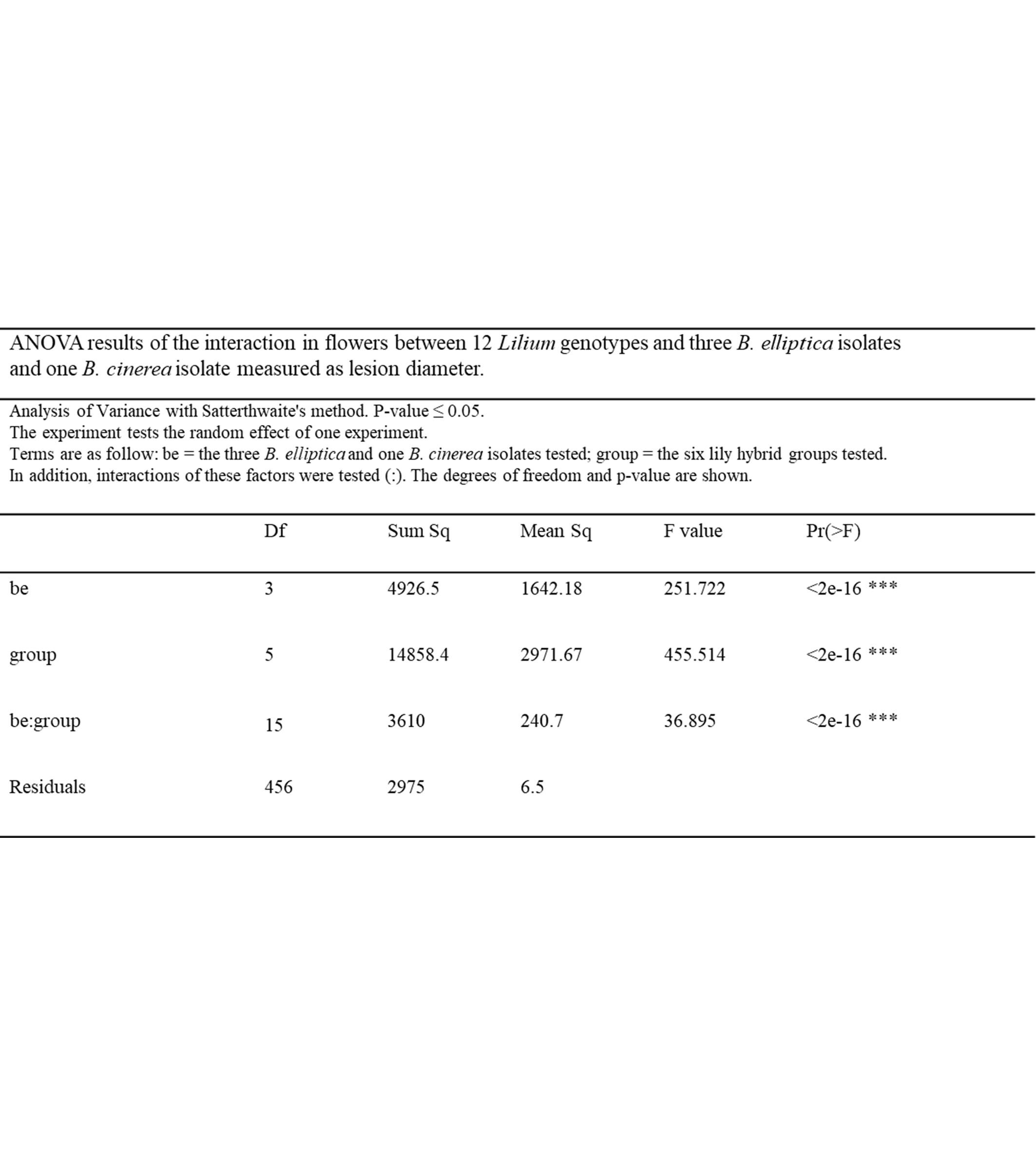

Supplement: Supplementary Figure 1 — (A) Fire blight symptoms observed upon and conidia inoculation of B. elliptica isolates Be9605 on leaf of lily cultivar OT-1 at 3 dpi. White dotted lines represent the chord of the ellipsoidal necrotic spots used to measure the lesion diameter. (B) Close-up of two representative necrotic lesions highlighted in the black quadrant in (A). Yellow arrows indicate area showing translucence, maceration softening and water soaking. Red arrows indicate necrotic collapsed tissue. [file Data_Sheet_1.zip › Supplementary Table S1.TIF]

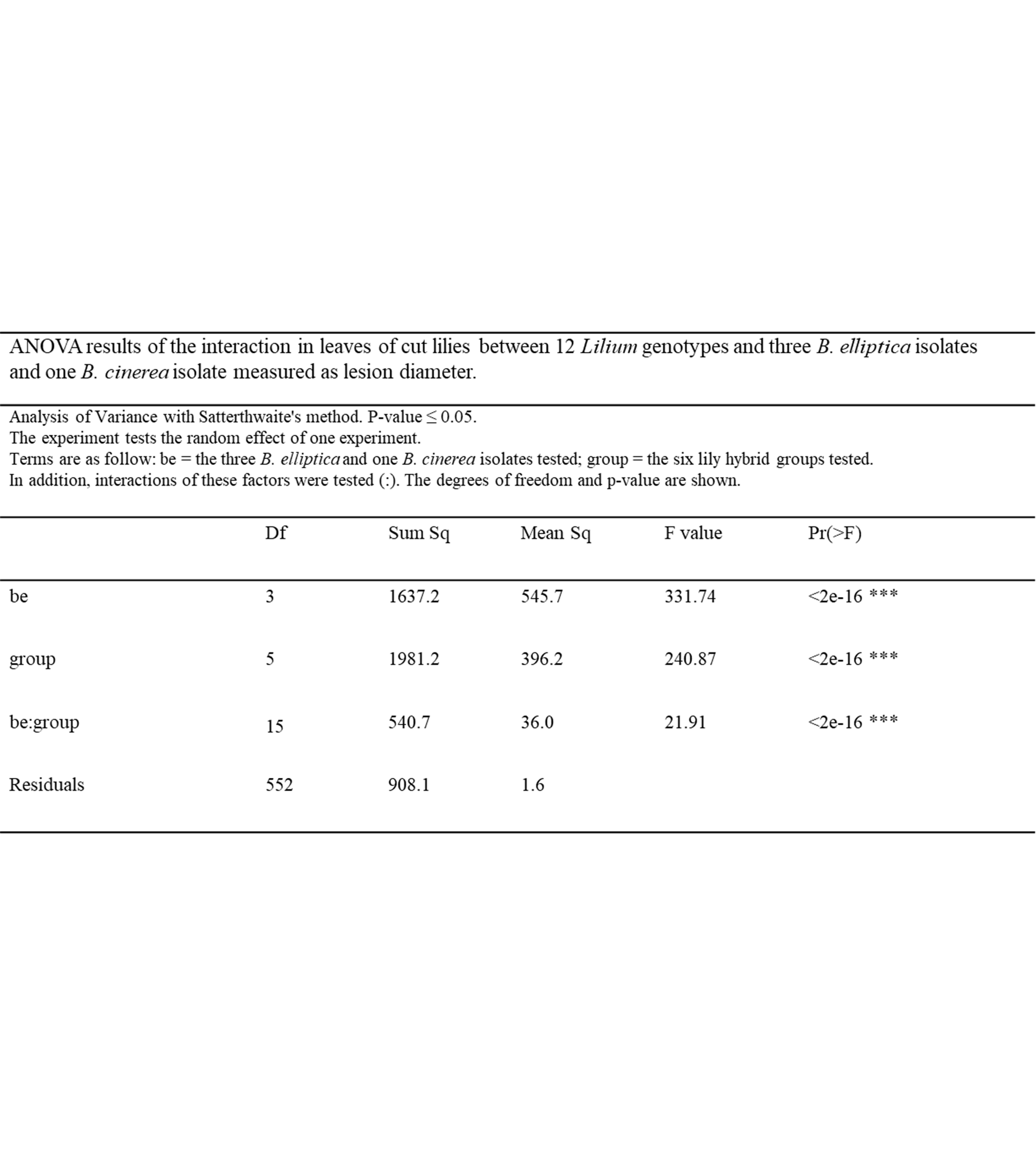

Supplement: Supplementary Figure 1 — (A) Fire blight symptoms observed upon and conidia inoculation of B. elliptica isolates Be9605 on leaf of lily cultivar OT-1 at 3 dpi. White dotted lines represent the chord of the ellipsoidal necrotic spots used to measure the lesion diameter. (B) Close-up of two representative necrotic lesions highlighted in the black quadrant in (A). Yellow arrows indicate area showing translucence, maceration softening and water soaking. Red arrows indicate necrotic collapsed tissue. [file Data_Sheet_1.zip › Supplementary Table S2.TIF]
